# Supplementary material for: Impact of Air Exposure Time on the Water Contact Angles of Daily Disposable Silicone Hydrogels
Source: Int J Mol Sci. 2019 Mar 15;20(6):1313. doi: 10.3390/ijms20061313 (PMC6471927; doi:10.3390/ijms20061313)
Supplement: Supplementary file 1 [file ijms-20-01313-s001.zip › Supplement_1 and Movies/Supplement_1.pdf]

Supplementary data

I. ANOVA and post-hoc comparisons of the dependencies of advancing (Fig. 1 in the main text) and receding (Fig. 3B in the main text) contact angles on time

In both cases the repeated measures ANOVA revealed highly significant ($P < 0.001$) difference between the group means. The results from the post-hoc multiple pairwise comparisons are summarized in Table S1 and S2.

Table S1. Probability values of multiple pairwise comparisons by Tukey-Kramer method between the advancing water contact angles of SiHy materials at different time points of desiccation/rehydration cycling ($p \leq 0.05$ is statistically significant).

Duration of cycling, h	DT vs AOD	DT vs MD	DT vs TE	MD vs AOD	MD vs TE	AOD vs TE
0	1.5×10^{-4}	1.5×10^{-4}	1.5×10^{-4}	1.5×10^{-4}	1.5×10^{-4}	0.01733
1	1.58×10^{-4}	1.58×10^{-4}	1.58×10^{-4}	1.58×10^{-4}	1.58×10^{-4}	1.58×10^{-4}
2	1.58×10^{-4}	1.58×10^{-4}	1.58×10^{-4}	1.58×10^{-4}	1.58×10^{-4}	1.58×10^{-4}
3	1.58×10^{-4}	1.58×10^{-4}	1.58×10^{-4}	1.58×10^{-4}	1.58×10^{-4}	1.58×10^{-4}
4	1.58×10^{-4}	1.58×10^{-4}	1.58×10^{-4}	1.58×10^{-4}	1.58×10^{-4}	1.4×10^{-4}
6	1.58×10^{-4}	1.58×10^{-4}	1.58×10^{-4}	1.58×10^{-4}	1.58×10^{-4}	1.12×10^{-3}
8	1.58×10^{-4}	1.58×10^{-4}	1.58×10^{-4}	1.58×10^{-4}	1.58×10^{-4}	0.063
10	1.58×10^{-4}	1.58×10^{-4}	1.58×10^{-4}	1.58×10^{-4}	1.58×10^{-4}	0.0023
12	1.58×10^{-4}	1.58×10^{-4}	1.58×10^{-4}	1.58×10^{-4}	1.58×10^{-4}	0.0012
14	1.58×10^{-4}	1.58×10^{-4}	1.58×10^{-4}	1.58×10^{-4}	1.58×10^{-4}	0.48
16	1.58×10^{-4}	1.58×10^{-4}	1.58×10^{-4}	1.58×10^{-4}	1.58×10^{-4}	0.065

Table S2. Probability values of multiple pair-wise comparisons by Tukey-Kramer method between the receding water contact angles of SiHy materials at different time points of desiccation/rehydration cycling ($p \leq 0.05$ is statistically significant).

Duration of cycling, h	DT vs AOD	DT vs MD	DT vs TE	MD vs AOD	MD vs TE	AOD vs TE
0	1.58×10^{-4}	1.58×10^{-4}	1.6×10^{-5}	0.017	1.58×10^{-4}	1.58×10^{-4}
12	1.5×10^{-3}	1.5×10^{-3}	1.58×10^{-4}	0.34	1.58×10^{-4}	1.58×10^{-4}
3	0.005	0.039	1.58×10^{-4}	0.01	1.58×10^{-4}	1.58×10^{-4}
4	2.7×10^{-3}	2×10^{-3}	1.58×10^{-4}	0.98	1.58×10^{-4}	1.58×10^{-4}
6	0.012	0.029	1.58×10^{-4}	0.36	1.58×10^{-4}	1.58×10^{-4}
8	3×10^{-3}	0.002	1.58×10^{-4}	0.98	1.58×10^{-4}	1.58×10^{-4}
10	1.58×10^{-4}	1.58×10^{-4}	1.58×10^{-4}	1.58×10^{-4}	1.58×10^{-4}	0.25
12	1.58×10^{-4}	1.58×10^{-4}	1.58×10^{-4}	1.58×10^{-4}	1.58×10^{-4}	0.32
14	1.58×10^{-4}	1.58×10^{-4}	1.58×10^{-4}	2×10^{-4}	1.58×10^{-4}	0.15
16	1.58×10^{-4}	1.58×10^{-4}	1.58×10^{-4}	1.58×10^{-4}	1.58×10^{-4}	0.38

II. Advancing contact angles of the contact lens measured with dynamic captive bubble methodology

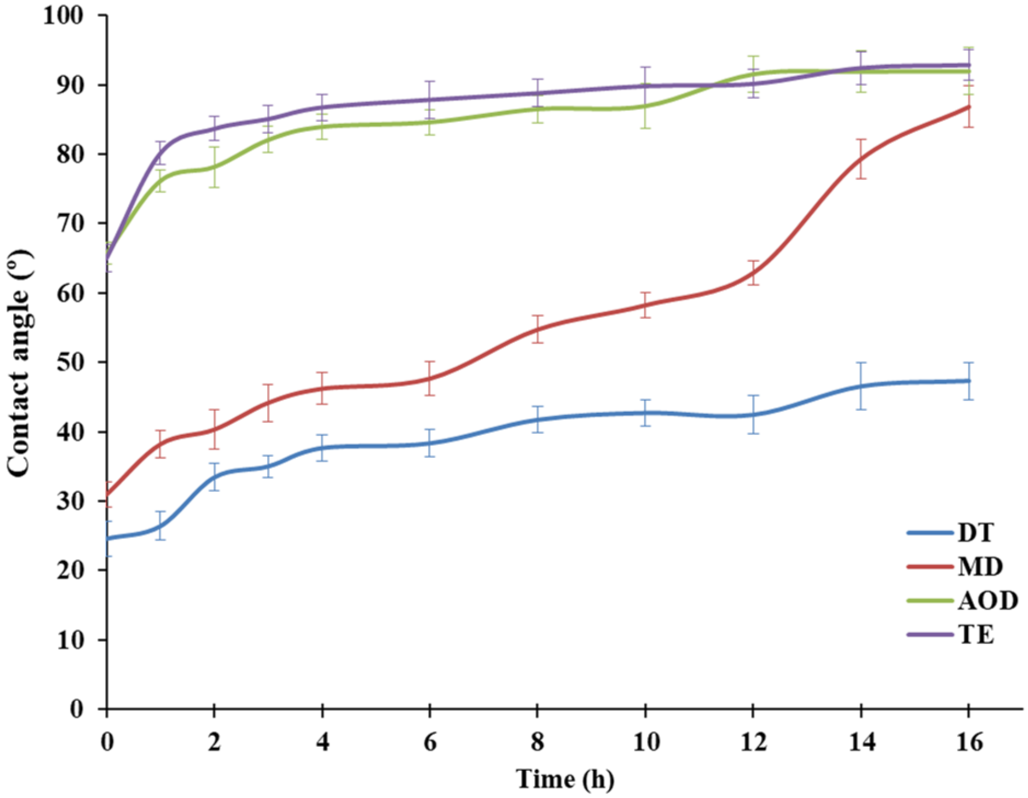

Fig. S1. Advancing contact angles determined by dynamic measurements with needle confined captive bubble. The data are very similar to the results obtained with sessile drop methodology, but contain higher noise (higher standard deviations). That is why sessile drop results were utilized in the main text.

III. Typical prelens tear film patterns *in vivo*

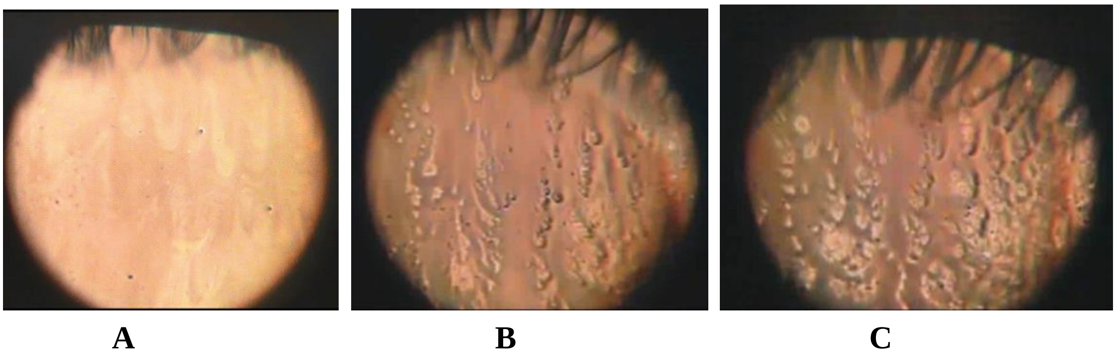

Fig. S2. Typical prelens tear film patterns observed with DR1-alpha specular microscope 15 s after eye opening in the eyes of healthy volunteers 2 h after fitting of DT or MD (panel A) or 2 h after fitting of AOD or TE (panels B and C). The data are based on at least five volunteers for each CL and are part of ongoing clinical observations which will be reported in separate study.
